# Hsp90β inhibition upregulates interferon response and enhances immune checkpoint blockade therapy in murine tumors

**DOI:** 10.3389/fimmu.2022.1005045

**Published:** 2022-10-20

**Authors:** Sharif Rahmy, Sanket J. Mishra, Sean Murphy, Brian S. J. Blagg, Xin Lu

**Affiliations:** ^1^ Department of Biological Sciences, Boler-Parseghian Center for Rare and Neglected Diseases, Harper Cancer Research Institute, University of Notre Dame, Notre Dame, IN, United States; ^2^ Integrated Biomedical Sciences Graduate Program, University of Notre Dame, Notre Dame, IN, United States; ^3^ Department of Chemistry and Biochemistry, Warren Family Research Center for Drug Discovery and Development, University of Notre Dame, Notre Dame, IN, United States; ^4^ Tumor Microenvironment and Metastasis Program, Indiana University Melvin and Bren Simon Comprehensive Cancer Center, Indianapolis, IN, United States

**Keywords:** heat shock protein 90 (hsp90), isoform-selective inhibitor, immune checkpoint blockade (ICB), prostate cancer, breast cancer, CDK4/6, interferon response, endogenous retrovirus

## Abstract

Response resistance to the immune checkpoint blockade (ICB) immunotherapy remains a major clinical challenge that may be overcome through the rational combination of ICB and specific targeted therapeutics. One emerging combination strategy is based on sensitizing ICB-refractory tumors with antagonists of 90kD heat shock protein (Hsp90) that target all four isoforms. However, pan-Hsp90 inhibitors are limited by the modest efficacy, on-target and off-tumor toxicities, and induction of the heat shock response (HSR) that overrides the effect of Hsp90 inhibition. Recently, we developed Hsp90β-selective inhibitors that were cytotoxic to cancer cells but did not induce HSR *in vitro*. Here, we report that the Hsp90β inhibitor NDNB1182 downregulated CDK4 (an Hsp90β-dependent client protein) and induced the expression of endogenous retroviral elements and interferon-stimulated genes. In syngeneic mouse models of prostate cancer and breast cancer, NDNB1182 significantly augmented the efficacy of ICB therapy. Furthermore, NDNB1182 showed superior tolerability to the pan-Hsp90 inhibitor Ganetespib in mice. Our findings provide evidence that Hsp90β inhibition is a potentially effective and safe regimen to combine with ICB to treat immunotherapy-refractory solid tumors.

## Introduction

Novel immunotherapies have revolutionized the treatment of cancer patients in recent years. The broadest impact comes from immune checkpoint blockade (ICB) that reinvigorates anti-tumor cytotoxic T lymphocytes (CTLs) using antibodies against CTLA4 or PD1/PD-L1 and generates therapeutic responses across a variety of cancer types (1). However, some solid tumor types remain largely resistant to ICB therapy, including breast cancer and prostate cancer which represent the first and third most frequently diagnosed malignancies worldwide, respectively (2). Advanced prostate cancer (metastatic and castration-resistant) shows overwhelming *de novo* resistance to anti-CTLA4 or anti-PD1 therapies (3–6). When ipilimumab and nivolumab were combined in a preliminary phase II clinical trial (CheckMate 650), improved objective response rates were reported (25% and 10% for pre- and post-chemotherapy), yet inadequate response rate and strong adverse effect remain frustrating (7). For metastatic triple-negative breast cancer, two ICB drugs, atezolizumab and pembrolizumab, in combination with chemotherapy, have been approved by the FDA to treat PD-L1^+^ cases (8, 9). However, the majority of the patients remain nonresponsive to ICB therapy. In order to bring broad benefits to patients with advanced prostate cancer and breast cancer, therapeutic approaches to overcome the resistance and sensitize the disease to ICB are urgently needed.

The 90kD heat shock protein (Hsp90) family functions as an evolutionarily conserved molecular chaperone to regulate protein homeostasis under physiological and stress conditions (10–13). Hsp90 family consists of four members: Hsp90α (inducible) and Hsp90β (constitutive) in the cytoplasm, glucose-regulated protein 94 (Grp94) in endoplasmic reticulum, and tumor necrosis factor receptor-associated protein-1 (Trap1) in mitochondria. Hsp90 proteins are responsible for the proper folding, disaggregation and intracellular trafficking of over 400 client proteins, including protein kinases, steroid hormone receptors, transcription factors, E3 ubiquitin ligases and more. Many of these clients control the hallmarks of cancer, therefore inhibition of Hsp90 may offer a unique benefit of co-targeting many oncogenic pathways (13). Various reports suggest that mutated and overexpressed oncoproteins rely more on the Hsp90 chaperone activity for proper folding, thus neoplastic cells may be more dependent on the function of Hsp90 chaperone for survival and proliferation than normal cells are, which could create a therapeutic window (14–17).

Many cell types express Hsp90 on the cell surface or secrete Hsp90 into the extracellular space (18, 19), and often Hsp90 expressed on the cell surface is more abundant in cancer cells than normal cells (20). Tumor cells appear to be more sensitive to Hsp90 inhibition, because the Hsp90 complex in cancer cells is distinct from normal cells with its higher affinity binding state (14). Moreover, in approximately 50% of cancers especially those fueled by MYC, Hsp90 acts as a nucleating site to form functionally integrated complexes termed ‘epichaperome’, which render tumor cells more sensitive to Hsp90 inhibitors (Hsp90i) (17). Lastly, Hsp90i preferentially accumulate in tumor cells as compared with normal cells (21–23). Therefore, Hsp90 is an attractive target for developing drugs to treat malignancies, including prostate cancer and breast cancer (24, 25).

Following the discovery of natural product inhibitors of Hsp90, geldanamycin (26) and radicicol (27), avid investment in the design, synthesis and evaluation of drug-like Hsp90i ensued (16, 28). So far, 18 small molecule drugs as pan-Hsp90 inhibitors (pan-Hsp90i) have entered clinical trials, but none has demonstrated satisfactory benefit-risk profile to be approved by the Food and Drug Administration (FDA) (29, 30). Pan-Hsp90i target the N-terminal domain (NTD) and bind competitively to the ATP binding site of all four Hsp90 isoforms. Challenges with current Hsp90i include limited efficacy, dose-limiting toxicities (DLTs), and various on-target and off-tumor toxicities. A major cause of the limited efficacy and DLTs is the induction of the pro-survival heat shock response (HSR) by pan-Hsp90 inhibition, because pan-Hsp90i trigger dissociation of heat shock factor 1 (Hsf1) from Hsp90 complex and Hsf1 subsequently enters nucleus and activates the transcription of Hsp27, Hsp40, Hsp70 and Hsp90 (31, 32), which counteracts the effect from the inhibitor. On the other hand, Hsp90i-associated hepatic, cardio and ocular toxicities may result from the disruption of clients. For example, the cardiac potassium channel human ether-a-go-go related gene (hERG) depends on Hsp90 for functional maturation, thus Hsp90i can cause deleterious effects on the hERG-related membrane potential (33). Interestingly, hERG is solely dependent on Hsp90α (34), suggesting that inhibition of Hsp90α (but not other isoforms) is likely to account more for the cardio and ocular toxicities. Moreover, Hsp90α and Hsp90β govern clientele and exert biological functions in non-redundant manners despite highly similar structures (35, 36), emphasizing the value of developing isoform-selective inhibitors. Using a structure-based molecular design and optimization approach, Blagg and colleagues developed various Grp94 inhibitors (37–40), Hsp90α inhibitors (Hsp90α-i) (41, 42), and Hsp90β inhibitors (Hsp90β-i) (43, 44). Among them, Hsp90β-i induce degradation of Hsp90β-dependent clients without concomitant degradation of Hsp90α clients or induction of the HSR (43, 44). Therefore, Hsp90β-i may overcome the obstacles encountered by pan-Hsp90i that have struggled in clinical trials.

Emerging evidence shows the involvement of Hsp90 in tumor immunity and the potential of enhancing immunotherapy with Hsp90i, although the mechanisms remain inadequately characterized and all these studies used pan-Hsp90i. For example, Ganetespib induced type I interferon response genes, such as interferon-induced protein with tetratricopeptide repeats (IFIT), through an unknown mechanism and promoted tumor cell killing by autologous T cells *in vitro* and anti-CTLA4 immunotherapy in mouse model of colorectal cancer (45). In addition, Ganetespib and 17-AAG decreased PD-L1 transcription through destabilizing Hsp90 clients MYC and STAT3 in monocytes and tumor cells at the sub-cytotoxic concentration, and Ganetespib diminished PD-L1 level on MC38 tumor cells *in vivo* (46). It is not yet explored whether isoform-selective Hsp90i, especially Hsp90β-i, can sensitize prostate cancer and breast cancer to immunotherapy, which is the central question for the current study to address.

## Results

### HSP90β-selective inhibitor NDNB1182 inhibits cell proliferation

**Figure 1:**
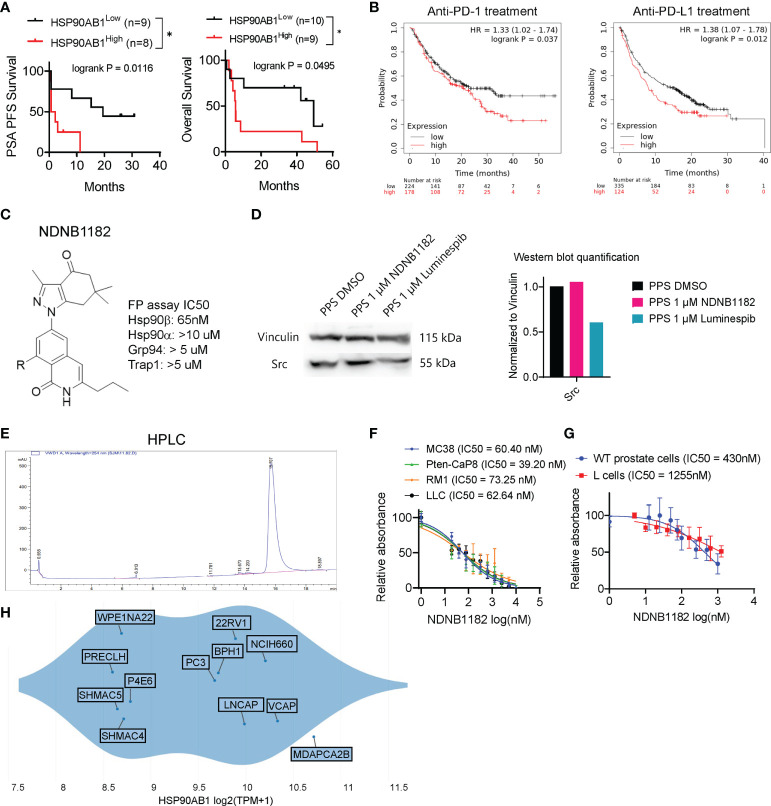
Hsp90β-selective inhibitor NDNB1182. **(A)** Association of high *HSP90AB1* expression with worse PSA progression free survival (PFS) and overall survival, with data extracted from the database of a phase 2 clinical trial of ipilimumab in metastatic castration-resistant prostate cancer *P<0.05, log rank test between the two cohorts (47). **(B)** Association of high *HSP90AB1* expression with worse overall survival for anti-PD1 or anti-PD-L1 treatment with data from multiple clinical studies, drawn with KMplot. **(C)** The generic structure of NDNB1182 and IC50 values against Hsp90 isoforms determined using fluorescence polarization (FP) assay. **(D)** Western blot of Src in mouse prostate cancer cell line PPS treated with DMSO, NDNB1182 or luminespib for 12 hours, with the semi-quantitative result presented on the right. **(E)** HPLC result confirming the high purity of NDNB1182. **(F)** Dose-response curves and IC50 values of NDNB1182 on four cancer cell lines. Nonlinear regression modeling with log(inhibitor) and normalized response of variable slopes was conducted in Graphpad Prism. **(G)** Dose-response curves and IC50 values of NDNB1182 on primary prostate epithelial cells grown from wild type C57BL/6 mice and the mouse fibroblast cell line L cells. **(H)** HSP90AB1 transcript levels for prostate-lineage cell lines in the Depmap database portal with the plot generated by Depmap.

We first conducted *in silico* analysis to support the proposition of inhibiting Hsp90β to enhance ICB therapy. From a phase 2 clinical trial of ipilimumab (anti-CTLA4) in 30 patients with metastatic castration-resistant prostate cancer (47), we retrieved the expression values of *HSP90AB1* (encoding Hsp90β) from the published RNA-seq data of the patient samples in the study (n=18) and separated the cohort to HSP90AB1^high^ and HSP90AB1^low^ groups. We compared the two groups for PSA progression free survival (PFS) and overall survival (OS). HSP90AB1^high^ group showed significantly worse outcome than the HSP90AB1^low^ group under ipilimumab therapy (**Figure 1A**). Next, we noticed that the KMplot tool recently gathered the survival data for patients across various cancer types treated with ICB therapies (48), so we used KMplot to compare the OS of HSP90AB1^high^ and HSP90AB1^low^ patients for either anti-PD1 treatment or anti-PD-L1 treatment. In both regimens, patients with HSP90AB1^high^ tumors showed significantly shorter OS (**Figure 1B**).

The Blagg laboratory recently reported the structure-based rational development of the 2H-isoquinolin-1-one based series of Hsp90β-i (44). To enhance the solubility of these Hsp90β-i compounds for *in vivo* application, Blagg’s group replaced the solvent exposed fragment (cyclohexanolamine) to reduce intermolecular pi-stacking, and synthesized the newest Hsp90β-i, NDNB1182 (**Figure 1C**) (Mishra et al, manuscript under preparation). Like other 2H-isoquinolin-1-one series of Hsp90β-i, NDNB1182 was evaluated for the binding affinities against the four Hsp90 isoforms by measuring the ability to competitively displace FITC-labeled geldanamycin (a pan-Hsp90i) in a fluorescence polarization (FP) assay (44, 49). NDNB1182 exhibited improved selectivity (over 150-fold) for the Hsp90β isoform over the highly identical Hsp90α isoform (**Figure 1C**). While pan-HSP90i luminespib reduced expression of the HSP90α-selecitve client protein Src (42, 50), NDNB1182 did not alter Src levels (**Figure 1D**). The purity of the compound was confirmed to be >95% by high-performance liquid chromatography (HPLC) (**Figure 1E**). We performed the resazurin assay to evaluate the cytotoxicity of NDNB1182 against murine colorectal cancer cell line MC38, murine prostate cancer cell lines Pten-CaP8 and RM1, and murine Lewis lung carcinoma LLC. The IC50 values for all the lines fell below 100nM and did not show significant differences among the lines (P = 0.0889, **Figure 1F**). As comparison, the effect of NDNB1182 was tested on two normal cell types, mouse prostate primary epithelial cells (grown from the dissociated prostate glands of wild type C57BL/6 mice) and the mouse fibroblast cell line L cells (ATCC, CRL-2648). IC50 of NDNB1182 was 430nM for normal prostate cells and 1255nM for L cells (**Figure 1G**), which were significantly higher than the IC50 values of the four mouse prostate cancer cell lines. Next, we plotted HSP90AB1 expression levels in various human prostate lineage cell lines at the Depmap portal. The result showed that various prostate cancer tissue-derived cell lines (MDAPCA2B, VCAP, NCIH660, LNCAP, 22Rv1, PC3) expressed HSP90AB1 at higher levels than the normal-like or ectopically transformed prostate cell lines (PRECLH, WPE1NA22, P4E6, SHMAC4, SHMAC5) (**Figure 1H**). Overall, these results support that Hsp90β is a promising target for prostate cancer and suggest that NDNB1182 selectively targets Hsp90β.

### NDNB1182 upregulates IFIT and endogenous retroviral element expression

**Figure 2:**
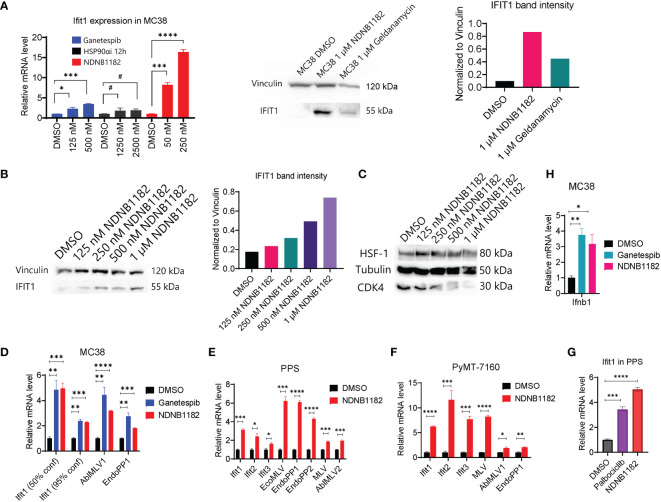
NDNB1182 upregulates IFIT and endogenous retroviral element expression. **(A)** qRT-PCR and western blot of Ifit1 expression in MC38 treated with DMSO, Ganetespib, NDNB1182, or Hsp90α-i 12h, at the indicated concentrations for 6 hours. Cells were at 30% confluence when treated with the inhibitors. **(B, C)** Western blot of IFIT1, HSF1 and CDK4 in DU145 treated with DMSO or a concentration gradient of NDNB1182 for 12 hours. In **(A, B)**, the quantification of band intensities was from one representative result of three repeated experiments that showed consistent results. **(D–F)** qRT-PCR result of *Ifit* and ERV elements in MC38, PPS and PyMT-7160 treated with DMSO, Ganetespib (1µM) or NDNB1182 (1µM) for 6 hours. In **(D)**, MC38 confluence during the inhibitor treatment was indicated. **(G)** qRT-PCR result of *Ifit1* expression in PPS treated with DMSO, NDNB1182 (1µM) and Palbociclib (2µM) for 6 hours. **(H)** qRT-PCR result of *Ifnb1* expression in MC38 (50% confluence) treated with DMSO, Ganetespib (1µM) or NDNB1182 (1µM) for 6 hours. In **(A, D–H)**, data represent mean ± SEM, N=3. #, P>0.05; *P<0.05, **P<0.01, ***P<0.001, ****P<0.0001, Student’s t-test.

The pan-Hsp90i Ganetespib was reported to induce IFIT1 expression at 125nM and higher (45). To examine whether isoform-selective Hsp90α-i or Hsp90β-i has this activity, we treated MC38 with Ganetespib, Hsp90α-i 12h (42) and NDNB1182 for 6 hours. Hsp90α inhibition induced no increase in *Ifit1* expression, whilst Ganetespib and NDNB1182 both significantly increased *Ifit1* expression with NDNB1182 inducing *Ifit1* by a higher magnitude (**Figure 2A**). We further confirmed the dose-dependent induction of IFIT1 expression by NDNB1182 in human prostate cancer cell line DU145 (**Figure 2B**).

Mbofung et al. did not identify the mechanism for Ganetespib to induce interferon-stimulated genes (ISGs), which is addressed in our study. We previously showed that Hsp90β-i compounds led to degradation of Hsp90β-dependent clients including CDK4 but did not trigger the undesired HSR such as HSF1 upregulation (43, 44). NDNB1182 was confirmed to induce a dose-dependent decline of CDK4 expression, but no increase of HSF1 (**Figure 2C**). Our result reinforced that CDK4/6 are Hsp90β-selective clients (51, 52). Interestingly, CDK4/6 inhibition was shown to reduce the expression of DNMT1 (encoding DNA methyltransferase 1), an E2F target gene, resulting in hypomethylation of the genome and activation of the expression of endogenous retroviral (ERV) elements and ISGs to enhance tumor antigen presentation and ultimately anti-tumor immunity (53). Taking these together, we hypothesized that NDNB1182 could achieve an equivalent effect as CDK4/6 inhibitor (like Palbociclib) to stimulate ERV and anti-viral ISG expression. To test this, we treated MC38 with Ganetespib and NDNB1182 and examined the expression of a list of murine ERV elements (54). Both inhibitors stimulated the levels of *Ifit1* and ERV genes AblMLV1 and EndoPP1 (**Figure 2D**). Interestingly, we observed that lower confluence of MC38 cells corresponded to higher Ifit1 induction by both Hsp90 inhibitors (**Figures 2A, D** **)**. This observation may be related to the DNA methylation dynamics during cell cycle (see Discussion). We expanded the treatment and detection to more syngeneic cell lines: the prostate cancer cell line PPS that we developed from the *PB-Cre^+^ Pten^L/L^ p53^L/L^ Smad4^L/L^* transgenic mouse model (55), and a mammary cancer cell line PyMT-7160 that we established in this study from the MMTV-PyMT (mouse mammary tumor virus promoter driven polyoma middle T-antigen) transgenic mice with autochthonous mammary adenocarcinoma (56). In both cell lines, NDNB1182 induced the expression of *Ifit* genes and various ERV elements (AblMLV1/2, EcoMLV, EndoPP1/2, MLV), although the exact fold changes differed (**Figures 2E, F**). Furthermore, both NDNB1182 and Palbociclib induced *Ifit1* expression in PPS (**Figure 2G**). *Ifnb1* (encoding interferon β1, a major type I interferon) was upregulated significantly in MC38 cells treated with Ganetespib and NDNB1182 (**Figure 2H**). These results demonstrate that NDNB1182, by inhibiting Hsp90β but not other isoforms, can downregulate CDK4/6 expression, activate ERV elements and stimulate the interferon response in cancer cells. This trait prompted us to test whether NDNB1182 could enhance ICB therapy.

### NDNB1182 enhanced ICB efficacy in the PPS syngeneic prostate cancer model

**Figure 3:**
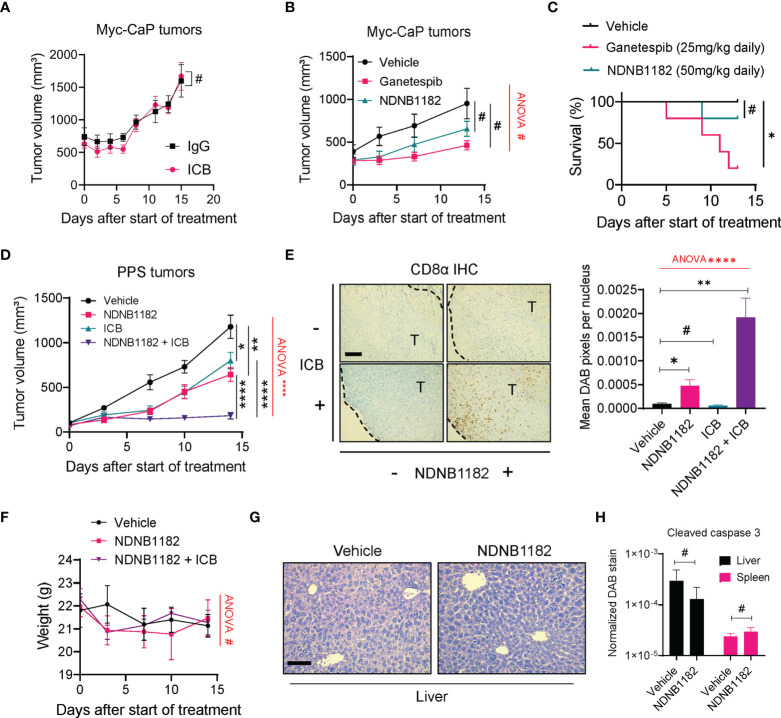
NDNB1182 enhanced ICB efficacy in the PPS syngeneic prostate cancer model. **(A)** Growth curves of Myc-CaP tumors treated with isotype IgG or ICB (anti-PD1 plus anti-CTLA4) administered intraperitoneally (i.p.) at 10mg/kg, twice/week. N=9/group. **(B)** Growth curves of Myc-CaP tumors treated with vehicle, Ganetespib (25mg/kg, i.p. daily) or NDNB1182 (50mg/kg, i.p. daily). N=10 tumors/group at the start of the treatment. **(C)** Survival curves for mice bearing Myc-CaP tumors and treated with vehicle, Ganetespib or NDNB1182. N=5/group. **(D)** Growth curves of PPS tumors treated with vehicle control (N=6), NDNB1182 (50mg/kg, i.p. daily; N=6), ICB (anti-PD1 plus anti-CTLA4, 10mg/kg, i.p., twice/week; N=10), or concurrent NDNB1182 plus ICB (N=8). In **(A, B, D)**, the randomized groups on day 0 of the treatment showed no significant difference based on statistical tests (for **(A)** two groups at day 0, P=0.8238 based on Mann Whitney test; for **(B)** three groups at day 0, P=0.4265 based on ANOVA test; for **(D)** four groups at day 0, P= 0.5561 based on ANOVA test). **(E)** Representative images and quantification results for CD8α immunohistochemistry on PPS tumors treated with the four regimens. The tumor regions (“T”) and the margin contours are denoted. N=5 for each condition. Image analysis and quantification was performed with ImageJ following a published protocol (59), where DAB signals were normalized to the number of nuclei present in the images. Scale bar 200µm. **(F)** Body weight changes of mice treated with vehicle, NDNB1182, or NDNB1182 plus ICB. **(G)** Representative H&E staining images of liver from vehicle-treated and NDNB1182-treated mice. Scale bar 50µm. **(H)** Quantification results for cleaved caspase 3 immunohistochemistry on liver and spleen tissues from vehicle-treated and NDNB1182-treated mice. In **(A–H)**, data represent mean ± SEM. #P>0.05; *P<0.05, **P<0.01, ****P<0.0001, Student’s t-test. In **(B, D–F)**, one-way ANOVA test result was shown in red (#P>0.05; ****P<0.0001). In **(C)**, #P>0.05; *P<0.05, logrank test.

We tested the anti-tumor effect of NDNB1182 in two prostate cancer syngeneic models, Myc-CaP and PPS. Myc-CaP was derived from c-myc transgenic mice in the FVB/N background (57) and responded poorly to ICB therapy (58). We confirmed that Myc-CaP failed to shrink under anti-PD1 plus anti-CTLA4 ICB treatment (**Figure 3A**). In this model, neither NDNB1182 (dosed at 50mg/kg, daily) nor Ganetespib (dosed at 25mg/kg, daily) had a significant impact on tumor growth (**Figure 3B**). Nevertheless, this model provided promising results for the toxicity profile of NDNB1182, because in the same experiment while Ganetespib showed significant toxicity leading to 80% (4 out of 5) mortality within 13 days of treatment, mice treated with NDNB1182 at two-fold of the dose showed no significant change of survival (**Figure 3C**).

PPS tumors grown in C57BL/6 background responded partially to ICB monotherapy (60). We treated PPS-carrying animals with vehicle control, NDNB1182, ICB (anti-PD1 + anti-CTLA4), or combination of NDNB1182 and ICB. Therapy with NDNB1182 or ICB each showed partial response, but the combination achieved remarkably enhanced efficacy (**Figure 3D**). Immunohistochemistry (IHC) staining of CD8α demonstrated that NDNB1182 alone induced a moderate increase of CD8^+^ T cell infiltration to the tumors, ICB alone had no effect, but the NDNB1182 plus ICB combination dramatically augmented the infiltration of CD8^+^ T cells (**Figure 3E**). Importantly, the animal body weight was not affected by NDNB1182 or NDNB1182 plus ICB treatments, showing low toxicity by NDNB1182 monotherapy or the combination treatment (**Figure 3F**). Furthermore, vehicle and NDNB1182 treated mice showed no discernable histological differences in the liver (**Figure 3G**) or the spleen (data not shown). NDNB1182 also caused no increase in apoptosis (as indicated by positive cleaved caspase 3 staining) in the liver or spleen (**Figure 3H**). These results provide further support for the safety of NDNB1182 at the dosage used.

The results from Myc-CaP and PPS models connect the anti-tumor activity of NDNB1182 with the ICB response of the models and illustrate the potential of enhancing ICB therapy with Hsp90β inhibition.

### NDNB1182 enhanced ICB therapy in the PyMT-7160 syngeneic mammary tumor model

**Figure 4:**
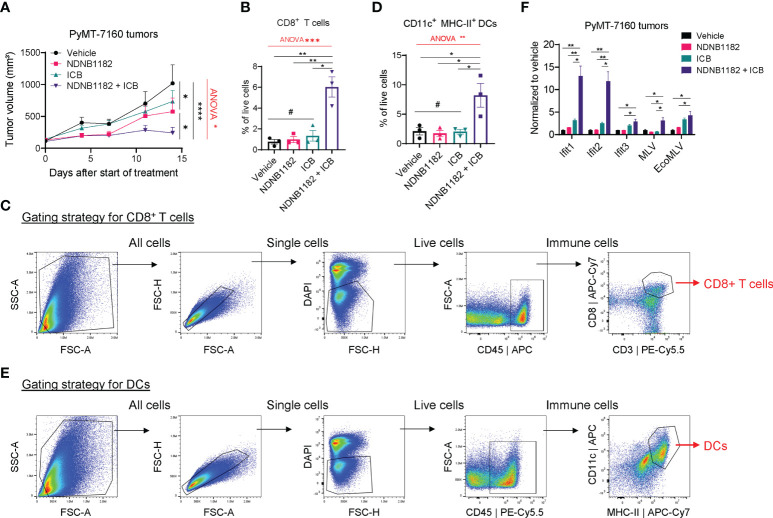
NDNB1182 enhanced ICB therapy in the PyMT-7160 syngeneic mammary tumor model. **(A)** Growth curves of PyMT-7160 tumors treated with vehicle control (N=8), NDNB1182 (50mg/kg, i.p. daily; N=10), ICB (anti-PD1 plus anti-CTLA4, 10mg/kg, i.p., twice/week; N=10), or concurrent NDNB1182 plus ICB (N=8). **(B, C)** Quantification results and gating strategy for tumor-infiltrating CD8^+^ T cells in treated PyMT-7160 tumors. **(D, E)** Quantification results and gating strategy for tumor-infiltrating CD11c^+^ MHC-II^+^ dendritic cells (DCs) in treated PyMT-7160 tumors. **(F)** qRT-PCR result of Ifit1/2/3 and two ERV elements for PyMT-7160 tumors treated with DMSO, NDNB1182, ICB, or NDNB1182 plus ICB (N=3/group).In **(A, B, D, F)**, data represent mean ± SEM. #P>0.05; *P<0.05, **P<0.01, ****P<0.0001, Student’s t-test. In **(A, B, D)**, one-way ANOVA test result was shown in red (*P<0.05, **P<0.01, ***P<0.001).

To test the activity of NDNB1182 to enhance immunotherapy in another tumor type, we orthotopically injected FVB/N female mice with PyMT-7160 mammary cancer cells and implemented a similar design of single and combination treatments. NDNB1182 and ICB each had moderate impact on tumor growth, but the NDNB1182 and ICB combination showed dramatic anti-tumor activity (**Figure 4A**). We dissociated the tumors at the endpoint and quantified the CTLs (CD45^+^ CD8^+^) and antigen-presenting dendritic cells (DCs, CD11c^+^ MHC-II^+^) with flow cytometry. We quantified CD11c^+^ MHC-II^+^ DCs because these cells may play an important role in presenting the tumor antigens to activate the T cell immunity. Consistent with the anti-tumor efficacy, only the NDNB1182 plus ICB treatment stimulated the tumor infiltration of both CTLs (**Figures 4B, C**) and DCs (**Figures 4D, E**) significantly compared with the control. We further confirmed that tumors treated with ICB plus NDNB1182 expressed *Ifit* genes and ERV elements MLV and EcoMLV at the highest level compared with other treatment regimens (**Figure 4F**). These results support that the combination of Hsp90β inhibition and ICB offers the most potent control on breast cancer progression through ERV and interferon response activation and anti-tumor immunity reprograming.

## Discussion

Most previous studies regarded Hsp90 chaperone as one machinery and inhibited Hsp90 activities in cancer models and clinical trials with pan-Hsp90i, which may largely account for the disappointing clinical performance of Hsp90 inhibition in cancer treatment so far. Because different Hsp90 isoforms have non-redundant functions executed by recognizing different sets of client proteins, targeting all isoforms is neither necessary nor beneficial. Here, we have challenged this paradigm by reporting, for the first time, the *in vivo* anti-tumor activity of Hsp90β-i and its potential as an immunotherapy sensitizer. Specifically, we made the following discoveries: (1) the new Hsp90β-i NDNB1182 demonstrated high selectivity toward Hsp90β over the other three Hsp90 isoforms and killed cancer cells at the lower 100nM IC50 level; (2) NDNB1182 induced the expression of various ERV elements and *Ifit* genes across different cancer cell lines; (3) NDNB1182 significantly augmented the efficacy from ICB therapy in the PPS prostate cancer and PyMT-7160 breast cancer models; (4) NDNB1182 displayed better tolerability than the pan-Hsp90i Ganetespib and did not cause mouse body condition deterioration or rapid weight loss when dosed at tumor-restricting levels.

Pan-Hsp90i as cancer monotherapy in clinical trials has encountered many challenges of limited efficacy and DLTs, leading to the dampened interest in oncological targeting of Hsp90 (29, 30). For example, a phase II trial using AUY922 in patients with metastatic gastrointestinal stromal tumor only showed modest anti-tumor effect but caused significant ocular toxicity (61). However, recent publications linking Hsp90 function with cancer immune modulation and showing how Hsp90 inhibition may enhance cancer immunotherapy in preclinical models have revived the interest in the combination of Hsp90i and ICB in the clinic (62, 63). For example, there are at least two ongoing Phase I trials that combine Hsp90i (XL888, TAS-116) and anti-PD1 in advanced gastrointestinal cancers (NCT03095781, NCT04999761). Nevertheless, the on-target toxicities from inhibiting all Hsp90 isoforms remain a concern and may lead to the difficulty in dose management of the combination treatment (63). This reasoning further highlights the significance of developing isoform-selective Hsp90i, which may still achieve the desired efficacy by targeting the isoform-dependent oncoprotein clients while avoiding the toxicities associated with the disruption of other clients, such as Hsp90α client hERG (34). To this end, we believe that Hsp90β emerges as the most promising isoform for the combinatorial targeting, because Hsp90β-i can induce degradation of Hsp90β-dependent clients that have well-established oncogenic functions (e.g. CDK4, CDK6, CXCR4, BRAF, HER2) without concomitant degradation of Hsp90α clients or induction of the HSR (43, 44). Our study confirms that NDNB1182 is effective *in vitro* and *in vivo* without causing discernable toxicities in mice (by contrast, Ganetespib was lethal even when administered at the half dose), electing NDNB1182 as a promising candidate for further preclinical validation and clinical development.

Our result showing that NDNB1182 activated the expression of ERV and IFIT genes is consistent with the previous finding with Ganetespib (45), suggesting the inhibition of Hsp90β among all four isoforms likely contributed the most to the activity of Ganetespib in the antiviral-like response in cancer cells. The CDK4 downregulation by NDNB1182 provides a logical mechanistic connection from Hsp90β inhibition to ERV and IFIT overexpression, because CDK4 is a client for Hsp90β and it is known that CDK4/6 inhibition can reactivate ERV expression and interferon response in cancer cells through downregulating DNMT1 expression and DNA methylation (53). CDK4/6 promotes Rb phosphorylation, leading to the release of E2F and subsequent transcriptional activation of E2F targets, including DNMT1. CDK4/6 inhibitors hinder the G1/S transition by inhibiting Rb phosphorylation and E2F release. Hsp90i may achieve the same effect by diminishing CDK4/6 protein level in the cells. Therefore, either by preventing Rb phosphorylation through CDK4/6 activity inhibition or by reducing the available pool of CDK4/6 through Hsp90 inhibition, the end result is a reduction in DNMT1 protein and subsequent DNA hypomethylation.

We noticed that the effect of NDNB1182 on Ifit1 induction was inversely related to cell confluence during the inhibitor treatment (**Figures 2A, D**). Because DNMT1 as the maintenance methyltransferase is responsible for the preservation of 5-methylcytosine in the genome during DNA replication (64), we speculate that a less confluent cell culture has higher portion of dividing cells than a much more confluent cell culture, therefore, the effect of NDNB1182 on DNA hypomethylation and *Ifit1* upregulation became more manifested in less confluent cells. Further experiments will help verify this proposition. In addition, we notice that the specific ERV elements activated by NDNB1182 differed among the cell lines in our study, and it is probably caused by the distinct epigenetic status of these ERV elements in these cell lines. Nonetheless, all the cell lines showed IFIT upregulation, thus producing equivalent interferon response signaling output to cooperate with immune checkpoint inhibition to reinvigorate T cell immunity. Indeed, efficacious immunotherapy depends on a potentiated type I interferon response (65, 66).

There are a few limitations in our study. First, the *in vivo* results were generated only using syngeneic models. While these models have served well as preclinical platforms for immunotherapy discovery and development, other types of cancer models such as genetically engineered mouse models and humanized mouse models with patient-derived tumors and reconstituted human immune system will provide important confirmative evidence. Second, the immunotherapy-sensitizer activity of NDNB1182 was only tested together with ICB therapy. Other immunotherapy modalities such as oncolytic virus and CAR-T therapy may also benefit from the combination with Hsp90β inhibition, especially in solid tumors. Third, our study does not exclude other immune modulatory mechanisms by Hsp90β inhibition besides the CDK4-ERV-IFIT axis that may also contribute to the combinatorial efficacy from NDNB1182 plus ICB treatment, which clearly warrants further investigations. Fourth, NDNB1182 was administered *via* intraperitoneal injection in our study, which would be inconvenient for the clinical application. Our teams are working on formulations that will allow oral delivery of NDNB1182 (or its improved analog). Lastly, given that hypomethylation and ERV may contribute to genomic instability in cancer (67, 68), the application of Hsp90β inhibition in clinical cancer therapy may not be suitable for certain patients (for example, young patients).

In conclusion, our results establish the preclinical evidence to support the rational combination of Hsp90β antagonists and immunotherapy in the treatment of intractable solid tumors, illuminating a clinical path for the better outcome of many cancer patients.

## Materials and methods

### Cell lines

Murine cell lines Pten-CaP8, RM1, LLC, and Myc-CaP and human cell line DU145 were purchased from ATCC and cultured in medium types recommended by ATCC. MC38 was purchased from kerafast and cultured in the recommended medium. PPS was developed from a spontaneous prostate tumor of *PB-Cre^+^ Pten^L/L^ p53^L/L^ Smad4^L/L^* transgenic mice (55). PyMT-7160 was developed from a spontaneous mammary tumor of the FVB/N-Tg(MMTV-PyVT)634Mul/J mice (Jackson Laboratory, 002374). PPS and PyMT-7160 were cultured in DMEM (GE Healthcare, SH30243.FS) supplemented with 10% fetal bovine serum (GE Healthcare, SH30396.03) and 100U/ml penicillin-streptomycin (Caisson Labs, PSL01). All the cell lines were cultured at 37°C in a humidified incubator with 5% CO2. All cells were tested for mycoplasma-free status using a Mycoplasma Assay Kit (Agilent Technologies, 302109).

### Chemicals

Hsp90α-i 12h (42) and Hsp90β-i NDNB1182 (Mishra et al, manuscript under preparation) were synthesized by the Blagg laboratory using the cited protocols. The identity of the chemicals was confirmed by high resolution mass spectrometry and nuclear magnetic resonance, while the purity of the compounds was confirmed to be >95% by high-performance liquid chromatography (HPLC). Ganetespib (MedChem Express, HY-15205) and Palbociclib (LC Laboratories, P-7788) were purchased.

### Mice

C57BL/6J males (Jackson Laboratory, 000664) and FVB/NJ males and females (Jackson Laboratory, 001800) were purchased at 5 weeks of age and used for experiments after one week of acclimation. All animals were maintained under pathogen-free conditions and cared for in accordance with the International Association for Assessment and Accreditation of Laboratory Animal Care policies and certification.

### Quantitative RT-PCR

RNA was isolated from cells and tissues using the EZ-10 Spin Column Total RNA Miniprep Kit (Bio Basic, BS1361) according to the manufacturer protocol. After RNA extraction, cDNA was synthesized using the All-in-one 5x RT MasterMix (ABM, G592). qPCR reactions were performed with the 2X SYBR Green Master mix (Bimake, B21203) and run on the CFX Connect Real-Time PCR Detection System (Bio-Rad, 1855201). *Gapdh* was used for normalization. Student’s t-test was performed based on the ΔΔC_T_ values. Unless otherwise specified, n=3 biological replicates per group were used for all qRT-PCR experiments. Primers were purchased from Eurofins Genomics. Primer sequences are listed in **Supplementary Table 1**.

### Western blot

Cell and tissue samples were lysed in RIPA buffer containing protease inhibitor (Bimake, B14012) and phosphatase inhibitor (Bimake, B15002). Protein concentration was determined using the Pierce BCA Protein Assay Kit (Thermo Scientific, 23225). After determining concentration, 30µg of protein lysate was boiled at 95°C for 5 minutes in Laemmli buffer (Bio-Rad, 161-0747), and subsequently run on SDS-PAGE. Gels were transferred to PDVF membranes (Bio-Rad, 1620177) using the Bio-Rad Trans-Blot Turbo Transfer System. After transfer, membranes were blocked for 1 hour in 5% fat free milk in TBS-T. After blocking, membranes were incubated in primary antibodies at concentrations according to manufacturer specifications for either 2 hours at room temperature or 16 hours at 4°C, washed three times in TBS-T, incubated for 1 hour at room temperature with HRP-linked secondary antibody (Cell Signaling Technologies, αRabbit cat#7074, αMouse cat#7076), and then washed again three times in TBS-T. Subsequently membranes were imaged using Clarity ECL (Bio-Rad, 1705060) on the ChemiDoc XRS+ imager (Bio-Rad, 1708265). The antibodies used are listed in **Supplementary Table 2**.

### Flow cytometry

Tumors were minced and digested in DMEM with 10% FBS and 1 mg/ml collagenase IV (STEMCELL Technologies, 07427) at 37°C for 1 h, followed by passing through 40μm strainers. Erythrocytes were depleted *via* hypotonic lysis. Cells were treated with mouse Fc-shield anti-CD16/CD32 (Tonbo Biosciences, 70-0161) for 15 minutes, and stained with primary fluorophore-conjugated antibodies for 30 minutes. Cells were washed twice and resuspended in a buffer containing DAPI (viability dye) and analyzed on the Beckman Coulter Cytoflex S cytometer. The antibodies used are listed in **Supplementary Table 2**.

### Immunohistochemistry

Tumors and livers/spleens were fixed in 10% neutral buffered formalin (VWR, 16004-128) for 24 hours and prepared as paraffin-embedded 5 µm sections. Antigen retrieval was performed using sodium citrate buffer (pH 6.0) at 95°C for 30 minutes followed by 115°C for 10 seconds. After antigen retrieval, endogenous peroxidase activity was blocked using 3% hydrogen peroxide for 10 minutes. Subsequently samples were blocked in 5% normal goat serum in TBS-T for 30 minutes and incubated with primary antibody anti-mouse CD8 (Cell Signaling Technology, 98941) or cleaved caspase 3 (Cell Signaling Technology, 9661) in a humidified chamber at 4°C for 16 hours. After washing, the VECTASTAIN Elite ABC-HRP Kit (Vector Laboratories, PK-6101) was used as secondary antibody and signal detection. Counterstain was performed using Mayer’s hematoxylin for 30 seconds. Imaging was performed using a Leica Aperio scanscope with 20× objective. At least 6 biological replicates were counted to quantify tumor-infiltrating CD8^+^ T cells or cleaved caspase 3 staining.

Hematoxilin and eosin stain (H&E)

Livers were fixed in 10% neutral buffered formalin for 24 hours and prepared as paraffin-embedded 5 µm sections. After rehydration, samples were stained for 8 minutes in Mayer’s hematoxilin. Excess dye was washed off in water for 8 minutes. Samples were immersed in 95% ethanol and stained in Eosin Y 1% (VWR, 101432-132) for 3 minutes. Excess dye was washed off in 95% ethanol after which slides were dehydrated and coverslipped and imaged using the Aperio scanscope.

### Tumor growth and inhibitor treatments

Syngeneic prostate tumor cell lines Myc-CaP or PPS was injected subcutaneously into 6-week-old FVB/NJ males or C57BL/6J males, respectively. Syngeneic mammary tumor cell line PyMT-7160 was injected to the mammary fat pads of 6-week-old FVB/NJ females. Tumors were measured with calipers and volumes were calculated using the formula length × width^2^ ÷ 2. Mice with tumors reaching the pre-specified volume range were randomized to receive the following therapies: anti-PD-1 (clone RMP1-14, Leinco Technologies, P362) and anti-CTLA4 (clone 9H10, Leinco Technologies, C1614) were injected intraperitoneally at 10mg/kg, twice per week; NDNB1182 or Ganetespib was dissolved in 10% DMSO, 40% polyethylene glycol 300, 5% Tween-80 and 55% ddH_2_O and injected intraperitoneally at 50mg/kg and 25mg/kg daily, respectively. To select the dosage of NDNB1182 for animal experiments, a maximum tolerated dose (MTD) pilot experiment was conducted in five 10-week old FVB/NJ mice with escalated dosages. After 10 days of daily treatment, the dose of 50mg/kg was the highest dose that caused no significant body weight loss or body condition deterioration, thus this dose was defined in our study as the desired dose to administer NDNB1182 in mice. For Ganetespib, 25mg/kg daily was used based on previous reports (69, 70). All treatments were continued until the specified experimental endpoints were reached.

### Statistical analyses

Statistical analyses were performed using GraphPad Prism v8.0. Unless otherwise mentioned, all data were presented as mean ± SEM (standard error of the mean). Sample sizes, error bars, P values, and statistical methods were denoted in the figures or figure legends. Statistical significance was defined as P < 0.05.

## Data availability statement

The original contributions presented in the study are included in the article/**Supplementary Material**. Further inquiries can be directed to the corresponding author.

## Ethics statement

The animal study was reviewed and approved by Institutional Animal Care and Use Committee at University of Notre Dame.

## Author contributions

SR: Conceptualization, investigation, methodology, data curation, formal analysis, validation, visualization, funding acquisition, writing – original draft. SJM: Resources. SM: Investigation. BB: Supervision. XL: Conceptualization, formal analysis, visualization, supervision, project administration, funding acquisition, writing – original draft, writing – review & editing. All authors contributed to the article and approved the submitted version.

## Funding

SR was supported by an Interdisciplinary Interface Training Program Grant from the Walther Cancer Foundation and Harper Cancer Research Institute at University of Notre Dame. XL was supported by National Institutes of Health grant R01CA248033, Department of Defense CDMRP PCRP grants W81XWH2010312 and W81XWH2010332, and Boler Family Foundation endowment at University of Notre Dame.

## Acknowledgments

We would like to thank the Lu lab members for constructive suggestions. We are grateful for the support from core facilities used in this study, especially Freimann Life Science Center and Tissue Repository at Harper Cancer Research Institute.

## Conflict of interest

The authors declare that the research was conducted in the absence of any commercial or financial relationships that could be construed as a potential conflict of interest.

## Publisher’s note

All claims expressed in this article are solely those of the authors and do not necessarily represent those of their affiliated organizations, or those of the publisher, the editors and the reviewers. Any product that may be evaluated in this article, or claim that may be made by its manufacturer, is not guaranteed or endorsed by the publisher.

## References

[1] Waldman AD Fritz JM Lenardo MJ. A guide to cancer immunotherapy: from T cell basic science to clinical practice. Nat Rev Immunol (2020) 20(11):651–68. doi: 10.1038/s41577-020-0306-5 PMC7238960 32433532

[2] Sung H Ferlay J Siegel RL Laversanne M Soerjomataram I Jemal A. Global cancer statistics 2020: GLOBOCAN estimates of incidence and mortality worldwide for 36 cancers in 185 countries. CA Cancer J Clin (2021). doi: 10.3322/caac.21660 33538338

[3] Topalian SL Hodi FS Brahmer JR Gettinger SN Smith DC McDermott DF. Safety, activity, and immune correlates of anti-PD-1 antibody in cancer. New Engl J Med (2012) 366(26):2443–54. doi: 10.1056/NEJMoa1200690 PMC3544539 22658127

[4] Kwon ED Drake CG Scher HI Fizazi K Bossi A van den Eertwegh AJM. Ipilimumab versus placebo after radiotherapy in patients with metastatic castration-resistant prostate cancer that had progressed after docetaxel chemotherapy (CA184-043): a multicentre, randomised, double-blind, phase 3 trial. Lancet Oncol (2014) 15(7):700–12. doi: 10.1016/S1470-2045(14)70189-5 PMC4418935 24831977

[5] Beer TM Kwon ED Drake CG Fizazi K Logothetis C Gravis G. Randomized, double-blind, phase III trial of ipilimumab versus placebo in asymptomatic or minimally symptomatic patients with metastatic chemotherapy-naive castration-resistant prostate cancer. J Clin Oncol (2017) 35(1):40–7. doi: 10.1200/jco.2016.69.1584 28034081

[6] Antonarakis ES Piulats JM Gross-Goupil M Goh J Ojamaa K Hoimes CJ. Pembrolizumab for treatment-refractory metastatic castration-resistant prostate cancer: Multicohort, open-label phase II KEYNOTE-199 study. J Clin Oncol (2020) 38(5):395–405. doi: 10.1200/jco.19.01638 31774688 PMC7186583

[7] Sharma P Pachynski RK Narayan V Fléchon A Gravis G Galsky MD. Nivolumab plus ipilimumab for metastatic castration-resistant prostate cancer: preliminary analysis of patients in the CheckMate 650 trial. Cancer Cell (2020) 38(4):489–99:e483. doi: 10.1016/j.ccell.2020.08.007 32916128

[8] Schmid P Adams S Rugo HS Schneeweiss A Barrios CH Iwata H. Atezolizumab and nab-paclitaxel in advanced triple-negative breast cancer. N Engl J Med (2018) 379(22):2108–21. doi: 10.1056/NEJMoa1809615 30345906

[9] Cortes J Cescon DW Rugo HS Nowecki Z Im SA Yusof MM. Pembrolizumab plus chemotherapy versus placebo plus chemotherapy for previously untreated locally recurrent inoperable or metastatic triple-negative breast cancer (KEYNOTE-355): a randomised, placebo-controlled, double-blind, phase 3 clinical trial. Lancet (2020) 396(10265):1817–28. doi: 10.1016/s0140-6736(20)32531-9 33278935

[10] Whitesell L Lindquist SL. HSP90 and the chaperoning of cancer. Nat Rev Cancer (2005) 5:761–72. doi: 10.1038/nrc1716 16175177

[11] Taipale M Jarosz DF Lindquist S. HSP90 at the hub of protein homeostasis: Emerging mechanistic insights. Nat Rev Mol Cell Biol (2010) 11(7):515–28. doi: 10.1038/nrm2918 20531426

[12] Schopf FH Biebl MM Buchner J. The HSP90 chaperone machinery. Nat Rev Mol Cell Biol (2017) 18(6):345–60. doi: 10.1038/nrm.2017.20 28429788

[13] Jaeger AM Whitesell L. HSP90: enabler of cancer adaptation. Annu Rev Cancer Biol (2019) 3:275–97. doi: 10.1146/annurev-cancerbio-030518-055533

[14] Kamal A Thao L Sensintaffar J Zhang L Boehm MF Fritz LC Burrows FJ A high-affinity conformation of Hsp90 confers tumour selectivity on Hsp90 inhibitors. Nature (2003) 425:407–10. doi: 10.1038/nature01913 14508491

[15] Chiosis G Neckers L. Tumor selectivity of Hsp90 inhibitors: the explanation remains elusive. ACS Chem Biol (2006) 1(5):279–84. doi: 10.1021/cb600224w 17163756

[16] Trepel J Mollapour M Giaccone G Neckers L. Targeting the dynamic HSP90 complex in cancer. Nat Rev Cancer (2010) 10(8):537–49. doi: 10.1038/nrc2887 PMC6778733 20651736

[17] Rodina A Wang T Yan P Gomes ED Dunphy MPS Pillarsetty N. The epichaperome is an integrated chaperome network that facilitates tumour survival. Nature (2016) 538(7625):397–401. doi: 10.1038/nature19807 27706135 PMC5283383

[18] Eustace BK Sakurai T Stewart JK Yimlamai D Unger C Zehetmeier C. Functional proteomic screens reveal an essential extracellular role for hsp90 alpha in cancer cell invasiveness. Nat Cell Biol (2004) 6(6):507–14. doi: 10.1038/ncb1131 15146192

[19] Sidera K Patsavoudi E. Extracellular HSP90: conquering the cell surface. Cell Cycle (2008) 7(11):1564–8. doi: 10.4161/cc.7.11.6054 18469526

[20] Tsutsumi S Scroggins B Koga F Lee MJ Trepel J Felts S. A small molecule cell-impermeant Hsp90 antagonist inhibits tumor cell motility and invasion. Oncogene (2008) 27(17):2478–87. doi: 10.1038/sj.onc.1210897 PMC2754825 17968312

[21] Vilenchik M Solit D Basso A Huezo H Lucas B He H. Targeting wide-range oncogenic transformation *via* PU24FCl, a specific inhibitor of tumor Hsp90. Chem Biol (2004) 11(6):787–97. doi: 10.1016/j.chembiol.2004.04.008 15217612

[22] Eiseman JL Lan J Lagattuta TF Hamburger DR Joseph E Covey JM. Pharmacokinetics and pharmacodynamics of 17-demethoxy 17-[[(2-dimethylamino)ethyl]amino]geldanamycin (17DMAG, NSC 707545) in C.B-17 SCID mice bearing MDA-MB-231 human breast cancer xenografts. Cancer Chemother Pharmacol (2005) 55(1):21–32. doi: 10.1007/s00280-004-0865-3 15338192

[23] Daozhen C Lu L Min Y Xinyu J Ying H. Synthesis of (131)I-labeled-[(131)I]iodo-17-allylamino-17-demethoxy geldanamycin ([(131)I]iodo-17-AAG) and its biodistribution in mice. Cancer Biother. Radiopharm. (2007) 22(5):607–12. doi: 10.1089/cbr.2006.363 17979563

[24] Azad AA Zoubeidi A Gleave ME Chi KN. Targeting heat shock proteins in metastatic castration-resistant prostate cancer. Nat Rev Urol (2015) 12(1):26–36. doi: 10.1038/nrurol.2014.320 25512207

[25] Birbo B Madu EE Madu CO Jain A Lu Y. Role of HSP90 in cancer. Int J Mol Sci (2021) 22(19):10317. doi: 10.3390/ijms221910317 34638658 PMC8508648

[26] Whitesell L Mimnaugh EG De Costa B Myers CE Neckers LM. Inhibition of heat shock protein HSP90-pp60v-src heteroprotein complex formation by benzoquinone ansamycins: essential role for stress proteins in oncogenic transformation. Proc Natl Acad Sci U.S.A. (1994) 91(18):8324–8. doi: 10.1073/pnas.91.18.8324 PMC44598 8078881

[27] Schulte TW Akinaga S Soga S Sullivan W Stensgard B Toft D. Antibiotic radicicol binds to the n-terminal domain of Hsp90 and shares important biologic activities with geldanamycin. Cell Stress Chaperones (1998) 3(2):100–8. doi: 10.1379/1466-1268(1998)003<0100:arbttn>2.3.co;2 PMC312953 9672245

[28] Neckers L Blagg B Haystead T Trepel JB Whitesell L Picard D. Methods to validate Hsp90 inhibitor specificity, to identify off-target effects, and to rethink approaches for further clinical development. Cell Stress Chaperones (2018) 23(4):467–82. doi: 10.1007/s12192-018-0877-2 PMC6045531 29392504

[29] Koren J 3rd Blagg BSJ. The right tool for the job: An overview of Hsp90 inhibitors. Adv Exp Med Biol (2020) 1243:135–46. doi: 10.1007/978-3-030-40204-4_9 32297216

[30] Sanchez J Carter TR Cohen MS Blagg BSJ. Old and new approaches to target the Hsp90 chaperone. Curr Cancer Drug Targets (2020) 20(4):253–70. doi: 10.2174/1568009619666191202101330 PMC7502213 31793427

[31] Bagatell R Paine-Murrieta GD Taylor CW Pulcini EJ Akinaga S Benjamin IJ. Induction of a heat shock factor 1-dependent stress response alters the cytotoxic activity of hsp90-binding agents. Clin Cancer Res (2000) 6(8):3312–8. 10955818

[32] Butler LM Ferraldeschi R Armstrong HK Centenera MM Workman P. Maximizing the therapeutic potential of HSP90 inhibitors. Mol Cancer Res (2015) 13(11):1445–51. doi: 10.1158/1541-7786.mcr-15-0234 PMC4645455 26219697

[33] Ficker E Dennis AT Wang L Brown AM. Role of the cytosolic chaperones Hsp70 and Hsp90 in maturation of the cardiac potassium channel HERG. Circ Res (2003) 92(12):e87–100. doi: 10.1161/01.res.0000079028.31393.15 12775586

[34] Peterson LB Eskew JD Vielhauer GA Blagg BS. The hERG channel is dependent upon the Hsp90α isoform for maturation and trafficking. Mol Pharm (2012) 9(6):1841–6. doi: 10.1021/mp300138n PMC3557513 22554505

[35] Voss AK Thomas T Gruss P. Mice lacking HSP90beta fail to develop a placental labyrinth. Development (2000) 127(1):1–11. doi: 10.1242/dev.127.1.1 10654595

[36] Grad I Cederroth CR Walicki J Grey C Barluenga S Winssinger N. The molecular chaperone Hsp90α is required for meiotic progression of spermatocytes beyond pachytene in the mouse. PloS One (2010) 5(12):e15770. doi: 10.1371/journal.pone.0015770 21209834 PMC3013136

[37] Duerfeldt AS Peterson LB Maynard JC Ng CL Eletto D Ostrovsky O. Development of a Grp94 inhibitor. J Am Chem Soc (2012) 134(23):9796–804. doi: 10.1021/ja303477g PMC3414055 22642269

[38] Muth A Crowley V Khandelwal A Mishra S Zhao J Hall J. Development of radamide analogs as Grp94 inhibitors. Bioorg. Med Chem (2014) 22(15):4083–98. doi: 10.1016/j.bmc.2014.05.075 PMC4142655 25027801

[39] Crowley VM Khandelwal A Mishra S Stothert AR Huard DJ Zhao J. Development of glucose regulated protein 94-selective inhibitors based on the BnIm and radamide scaffold. J Med Chem (2016) 59(7):3471–88. doi: 10.1021/acs.jmedchem.6b00085 PMC4979570 27003516

[40] Crowley VM Huard DJE Lieberman RL Blagg BSJ. Second generation Grp94-selective inhibitors provide opportunities for the inhibition of metastatic cancer. Chemistry (2017) 23(62):15775–82. doi: 10.1002/chem.201703398 PMC5722212 28857290

[41] Liu W Vielhauer GA Holzbeierlein JM Zhao H Ghosh S Brown D. KU675, a concomitant heat-shock protein inhibitor of Hsp90 and Hsc70 that manifests isoform selectivity for Hsp90α in prostate cancer cells. Mol Pharmacol (2015) 88(1):121–30. doi: 10.1124/mol.114.097303 PMC4468638 25939977

[42] Mishra SJ Khandelwal A Banerjee M Balch M Peng S Davis RE. Selective inhibition of the Hsp90α isoform. Angew Chem Int Ed Engl (2021) 60(19):10547–51. doi: 10.1002/anie.202015422 PMC8086817 33621416

[43] Khandelwal A Kent CN Balch M Peng S Mishra SJ Deng J. Structure-guided design of an Hsp90β n-terminal isoform-selective inhibitor. Nat Commun (2018) 9(1):425. doi: 10.1038/s41467-017-02013-1 29382832 PMC5789826

[44] Mishra SJ Liu W Beebe K Banerjee M Kent CN Munthali V. The development of Hsp90β-selective inhibitors to overcome detriments associated with pan-Hsp90 inhibition. J Med Chem (2021) 64(3):1545–57. doi: 10.1021/acs.jmedchem.0c01700 PMC8996186 33428418

[45] Mbofung RM McKenzie JA Malu S Zhang M Peng W Liu C. HSP90 inhibition enhances cancer immunotherapy by upregulating interferon response genes. Nat Commun (2017) 8(1):451. doi: 10.1038/s41467-017-00449-z 28878208 PMC5587668

[46] Zavareh RB Spangenberg SH Woods A Martínez-Peña F Lairson LL. HSP90 inhibition enhances cancer immunotherapy by modulating the surface expression of multiple immune checkpoint proteins. Cell Chem Biol (2021) 28(2):158–168.e155. doi: 10.1016/j.chembiol.2020.10.005 33113406

[47] Subudhi SK Vence L Zhao H Blando J Yadav SS Xiong Q. Neoantigen responses, immune correlates, and favorable outcomes after ipilimumab treatment of patients with prostate cancer. Sci Transl Med (2020) 12(537):eaaz3577. doi: 10.1126/scitranslmed.aaz3577 32238575

[48] Lánczky A Győrffy B. Web-based survival analysis tool tailored for medical research (KMplot): Development and implementation. J Med Internet Res (2021) 23(7):e27633. doi: 10.2196/27633 34309564 PMC8367126

[49] Kim J Felts S Llauger L He H Huezo H Rosen N. Development of a fluorescence polarization assay for the molecular chaperone Hsp90. J Biomol. Screen (2004) 9(5):375–81. doi: 10.1177/1087057104265995 15296636

[50] Millson SH Truman AW Rácz A Hu B Panaretou B Nuttall J. Expressed as the sole Hsp90 of yeast, the alpha and beta isoforms of human Hsp90 differ with regard to their capacities for activation of certain client proteins, whereas only Hsp90beta generates sensitivity to the Hsp90 inhibitor radicicol. FEBS J (2007) 274(17):4453–63. doi: 10.1111/j.1742-4658.2007.05974.x 17681020

[51] Verba KA Wang RY Arakawa A Liu Y Shirouzu M Yokoyama S. Atomic structure of Hsp90-Cdc37-Cdk4 reveals that Hsp90 traps and stabilizes an unfolded kinase. Science (2016) 352(6293):1542–7. doi: 10.1126/science.aaf5023 PMC5373496 27339980

[52] Wu X Yang X Xiong Y Li R Ito T Ahmed TA. Distinct CDK6 complexes determine tumor cell response to CDK4/6 inhibitors and degraders. Nat Cancer (2021) 2(4):429–43. doi: 10.1038/s43018-021-00174-z PMC8462800 34568836

[53] Goel S DeCristo MJ Watt AC BrinJones H Sceneay J Li BB. CDK4/6 inhibition triggers anti-tumour immunity. Nature (2017) 548(7668):471–5. doi: 10.1038/nature23465 PMC5570667 28813415

[54] Bock S Mullins CS Klar E Pérot P Maletzki C Linnebacher M. Murine endogenous retroviruses are detectable in patient-derived xenografts but not in patient-individual cell lines of human colorectal cancer. Front Microbiol (2018) 9:789. doi: 10.3389/fmicb.2018.00789 29755432 PMC5932414

[55] Lu X Horner JW Paul E Shang X Troncoso P Deng P. Effective combinatorial immunotherapy for castration-resistant prostate cancer. Nature (2017) 543(7647):728–32. doi: 10.1038/nature21676 PMC5374023 28321130

[56] Attalla S Taifour T Bui T Muller W. Insights from transgenic mouse models of PyMT-induced breast cancer: recapitulating human breast cancer progression *in vivo* . Oncogene (2021) 40(3):475–91. doi: 10.1038/s41388-020-01560-0 PMC7819848 33235291

[57] Watson PA Ellwood-Yen K King JC Wongvipat J Lebeau MM Sawyers CL. Context-dependent hormone-refractory progression revealed through characterization of a novel murine prostate cancer cell line. Cancer Res (2005) 65(24):11565–71. doi: 10.1158/0008-5472.can-05-3441 16357166

[58] Dudzinski SO Cameron BD Wang J Rathmell JC Giorgio TD Kirschner AN. Combination immunotherapy and radiotherapy causes an abscopal treatment response in a mouse model of castration resistant prostate cancer. J ImmunoTher. Cancer (2019) 7(1):218. doi: 10.1186/s40425-019-0704-z 31412954 PMC6694548

[59] Crowe AR Yue W. Semi-quantitative determination of protein expression using immunohistochemistry staining and analysis: An integrated protocol. Bio Protoc (2019) 9(24):e3465. doi: 10.21769/BioProtoc.3465 PMC6924920 31867411

[60] Guan X Polesso F Wang C Sehrawat A Hawkins RM Murray SE. Androgen receptor activity in T cells limits checkpoint blockade efficacy. Nature (2022) 606(7915):791–6. doi: 10.1038/s41586-022-04522-6 PMC10294141 35322234

[61] Chiang N-J Yeh K-H Chiu C-F Chen J-S Yen C-C Lee K-D. Results of phase II trial of AUY922, a novel heat shock protein inhibitor in patients with metastatic gastrointestinal stromal tumor (GIST) and imatinib and sunitinib therapy. J Clin Oncol (2016) 34(4_suppl):134–4. doi: 10.1200/jco.2016.34.4_suppl.134

[62] Graner MW. HSP90 and immune modulation in cancer. Adv Cancer Res (2016) 129:191–224. doi: 10.1016/bs.acr.2015.10.001 26916006

[63] Graner MW. Making HSP90 inhibitors great again? unite for better cancer immunotherapy. Cell Chem Biol (2021) 28(2):118–20. doi: 10.1016/j.chembiol.2021.02.002 33607004

[64] Hervouet E Nadaradjane A Gueguen M Vallette FM Cartron PF. Kinetics of DNA methylation inheritance by the Dnmt1-including complexes during the cell cycle. Cell Div (2012) 7:5. doi: 10.1186/1747-1028-7-5 22348533 PMC3307489

[65] Zitvogel L Galluzzi L Kepp O Smyth MJ Kroemer G. Type I interferons in anticancer immunity. Nat Rev Immunol (2015) 15(7):405–14. doi: 10.1038/nri3845 26027717

[66] Yu R Zhu B Chen D. Type I interferon-mediated tumor immunity and its role in immunotherapy. Cell Mol Life Sci (2022) 79(3):191. doi: 10.1007/s00018-022-04219-z 35292881 PMC8924142

[67] Campbell IM Gambin T Dittwald P Beck CR Shuvarikov A Hixson P. Human endogenous retroviral elements promote genome instability *via* non-allelic homologous recombination. BMC Biol (2014) 12:74. doi: 10.1186/s12915-014-0074-4 25246103 PMC4195946

[68] Sheaffer KL Elliott EN Kaestner KH. DNA Hypomethylation contributes to genomic instability and intestinal cancer initiation. Cancer Prev Res (Phila) (2016) 9(7):534–46. doi: 10.1158/1940-6207.Capr-15-0349 PMC4930698 26883721

[69] Lin TY Bear M Du Z Foley KP Ying W Barsoum J. The novel HSP90 inhibitor STA-9090 exhibits activity against kit-dependent and -independent malignant mast cell tumors. Exp Hematol (2008) 36(10):1266–77. doi: 10.1016/j.exphem.2008.05.001 PMC3837096 18657349

[70] Shimamura T Perera SA Foley KP Sang J Rodig SJ Inoue T. Ganetespib (STA-9090), a nongeldanamycin HSP90 inhibitor, has potent antitumor activity in *in vitro* and *in vivo* models of non-small cell lung cancer. Clin Cancer Res (2012) 18(18):4973–85. doi: 10.1158/1078-0432.Ccr-11-2967 PMC3477583 22806877

