# Supplementary material for: Hsp90β inhibition upregulates interferon response and enhances immune checkpoint blockade therapy in murine tumors
Source: Front Immunol. 2022 Oct 20;13:1005045. doi: 10.3389/fimmu.2022.1005045 (PMC9630337; doi:10.3389/fimmu.2022.1005045)
Supplement: Supplementary file 1 [file DataSheet_1.pdf]

Supplementary Material

Supplementary Table 1. Primer sequences used for qRT-PCR.

Gene	Abbreviation	Species	Sequence	Direction
Abelson (P160) murine leukemia virus	AblMlv1	Mouse	TGT ACG AGG GCG TTT GGA AG	F
Abelson (P160) murine leukemia virus	AblMlv1	Mouse	GGG TAC ACA CCC CTA GCA G	R
Abelson (P160) murine leukemia virus	AblMlv2	Mouse	CTG GGA GAA AGC GAT GCA CT	F
Abelson (P160) murine leukemia virus	AblMlv2	Mouse	CGC TCA TCT TCA TTT AGG CTG C	R
Ecotropic murine leukemia virus	EcoMlv	Mouse	GTA ACG CCA TTT TGC AAG GC	F
Ecotropic murine leukemia virus	EcoMlv	Mouse	ATG AGG CTT GGG GTT GAT CC	R
Mus musculus mobilized endogenous polytropic provirus	EndoPP1	Mouse	GGC CGG AAC ACG AGT AAG AG	F
Mus musculus mobilized endogenous polytropic provirus	EndoPP1	Mouse	TTG GCC ACC GAC TGA CTT AC	R
Mus musculus mobilized endogenous polytropic provirus	EndoPP2	Mouse	TCT ATA GTC CCT GAG ACT GCC C	F
Mus musculus mobilized endogenous polytropic provirus	EndoPP2	Mouse	CAA CCA GCA CTC TTG GGT TTT GT	R
Murine leukemia virus	Mlv	Mouse	GTA CCA ACA GGG TGT GGA GG	F

Murine leukemia virus	Mlv	Mouse	TTG ATG TCA CTG GAG ACC GC	R
Interferon-induced protein with tetratricopeptide repeats 1	IFIT1	Mouse	TCTGCTCTGCTGAAAACCCA	F
Interferon-induced protein with tetratricopeptide repeats 1	IFIT1	Mouse	CACCATCAGCATTCTCTCCCAT	R
Interferon-induced protein with tetratricopeptide repeats 2	IFIT2	Mouse	GTAGGGGTTACATCCGGCAC	F
Interferon-induced protein with tetratricopeptide repeats 2	IFIT2	Mouse	TCTGTGCAGCACCTCTAAGTC	R
Interferon-induced protein with tetratricopeptide repeats 3	IFIT3	Mouse	CTGAACTGCTCAGCCCACA	F
Interferon-induced protein with tetratricopeptide repeats 3	IFIT3	Mouse	TTCCCGGTTGACCTCACTCA	R
Interferon -β	Ifnb1	Mouse	ATCAACCTCACCTACAGGGC	F
Interferon -β	Ifnb1	Mouse	ATCTCTTGGATGGCAAAGGCA	R
Glyceraldehyde 3-phosphate dehydrogenase	GAPDH	Mouse	AGGTCGGTGTGAACGGATTTG	F
Glyceraldehyde 3-phosphate dehydrogenase	GAPDH	Mouse	TGTAGACCATGTAGTTGAGGTCA	R

Supplementary Table 2. Antibodies used in western blot and flow cytometry.

**Target**	**Clone**	**Manufacturer**	**Cat#**
HSP90	AC88	Enzo life sciences	ADI-SPA-830
Vinculin	V284	Millipore	05-386
CDK4	D9G3E	CST	12790
α-Tubulin	DM1A	Sigma-Aldrich	T9026
HSF-1	10H8	Enzo Life Sciences	ADI-SPA-950-D
IFIT1	OTI3G8	Origene	TA500948S
CD45	UCHL1	BD	561888
CD8a	53-6.7	BD	561095
CD3ε	17A2	Tonbo	65-0032
CD11c	N418	Tonbo	50-0114
MHC-II	M5/114.15.2	Tonbo	25-5321
SRC	36D10	CST	2019
CD8a	D4W2Z	CST	98941
Cleaved Caspase 3	269518	CST	9661
